# The SAMPL6 challenge on predicting octanol–water partition coefficients from EC-RISM theory

**DOI:** 10.1007/s10822-020-00283-4

**Published:** 2020-01-24

**Authors:** Nicolas Tielker, Daniel Tomazic, Lukas Eberlein, Stefan Güssregen, Stefan M. Kast

**Affiliations:** 1 Physikalische Chemie III, Technische Universität Dortmund, Otto-Hahn-Str. 4a, 44227 Dortmund, Germany; 2 Sanofi-Aventis Deutschland GmbH, R&D Integrated Drug Discovery, 65926 Frankfurt am Main, Germany

**Keywords:** SAMPL6, Solvation model, Quantum chemistry, Integral equation theory, EC-RISM, log *P*

## Abstract

Results are reported for octanol–water partition coefficients (log *P*) of the neutral states of drug-like molecules provided during the SAMPL6 (Statistical Assessment of Modeling of Proteins and Ligands) blind prediction challenge from applying the “embedded cluster reference interaction site model” (EC-RISM) as a solvation model for quantum-chemical calculations. Following the strategy outlined during earlier SAMPL challenges we first train 1- and 2-parameter water-free (“dry”) and water-saturated (“wet”) models for *n*-octanol solvation Gibbs energies with respect to experimental values from the “Minnesota Solvation Database” (MNSOL), yielding a root mean square error (RMSE) of 1.5 kcal mol^−1^ for the best-performing 2-parameter wet model, while the optimal water model developed for the p*K*_a_ part of the SAMPL6 challenge is kept unchanged (RMSE 1.6 kcal mol^−1^ for neutral compounds from a model trained on both neutral and ionic species). Applying these models to the blind prediction set yields a log *P* RMSE of less than 0.5 for our best model (2-parameters, wet). Further analysis of our results reveals that a single compound is responsible for most of the error, SM15, without which the RMSE drops to 0.2. Since this is the only compound in the challenge dataset with a hydroxyl group we investigate other alcohols for which Gibbs energy of solvation data for both water and *n*-octanol are available in the MNSOL database to demonstrate a systematic cause of error and to discuss strategies for improvement.

**Electronic supplementary material:**

The online version of this article (10.1007/s10822-020-00283-4) contains supplementary material, which is available to authorized users.

## Introduction

The prediction of physicochemical properties of small, drug-like molecules has been the focus of the Statistical Assessment of the Modeling of Proteins and Ligands series of challenges for several years [1]. In the latest instance, a subset of the molecules provided during the SAMPL6 challenge for the prediction of acidity constants (p*K*_a_) [2, 3] was selected by the organizers to challenge the community again with the task to predict their neutral-state partitioning thermodynamics measured by the octanol–water partition coefficients, log *P* [4]. Compared to the p*K*_a_ prediction challenge the resulting tasks partly overlap (solvation properties in an aqueous phase, adequate treatment of tautomeric or “microstates”), but the problem of partition coefficients, translated into the difference of solvation Gibbs (free) energies, implies additional problems. In contrast to the previous SAMPL5 challenge on cyclohexane-water distribution coefficients (log *D* at a given aqueous pH) [5, 6] the problem is simpler as no ionic species have to be accounted for, but a non-aqueous polar solvent such *n*-octanol poses an additional difficulty as neglecting or accounting for the experimentally known water content of 48.91 mg g^−1^ at a temperature of 298.15 K [7] could have significant impact on the accuracy of the predictions.

As in the earlier challenges we here employed the “embedded cluster reference interaction site model” (EC-RISM) to characterize the thermodynamics of the solvation process [8]. This method combines 3D RISM integral equation theory [9–11] with a quantum-chemical (QC) description of the solute to capture electronic solute polarization upon entering a polar solvent environment. This is achieved by calculating the solvent distribution functions around the solute mapped onto background charges around the solute. These are applied in the QC calculations from which, after convergence of an iterative cycle, the wave function of the solute in solution as well as the excess chemical potential at infinite dilution and other properties of the fully polarized solute can be determined [12, 13]. As usual, we took the sum of the polarized electronic energy and the excess chemical potential as an estimate of the Gibbs energy of the molecule in solution to calculate derived properties such as solvation Gibbs energies (by referencing to a gas phase calculation), acidity constants, partition and distribution coefficients, or tautomer and conformational populations of molecules under ambient and extreme conditions in a variety of solvents [14–21].

Because the known errors resulting from the approximations made in 3D RISM theory have been shown to scale with the partial molar volume (PMV) of the molecule [22–24] that can also be determined from 3D RISM calculations, we have already successfully trained and applied corrections for EC-RISM using only two free parameters for small-molecule solvation Gibbs energies in water and cyclohexane [3, 6]. Similar to Ref. [25] (which was restricted to force field-based 3D RISM log *P* calculations ignoring electronic polarization), this scheme was here extended to “dry” *n*-octanol and “wet” saturated *n*-octanol–water mixtures to model the organic phase. We here adhered to our physically “conservative” strategy to train models on “basic” quantities such as solvation Gibbs energies only. This way we avoid overfitting and are able to measure the theoretical model performance directly which facilitates systematic optimization on the premise that any derived quantity (such as a partition coefficient) should automatically improve as well. Moreover, in contrast to the case of predicting acidity constants where a second set of empirical corrections (slope parameter and additive constant related to the Gibbs energy of the proton [3, 6, 26]) was applied we can here determine the partition coefficient directly from the corrected Gibbs energies in the respective solvents, making this an even stronger test case for the validity of the PMV correction, since any potentially existing deficiencies cannot be alleviated by the second correction.

After a brief introduction into methods and computational aspects which can be found in full detail in the our earlier SAMPL challenge papers [3, 6], we outline model training for the calculation of solvation Gibbs energies of molecules in dry and wet *n*-octanol with respect to experimental values taken from the Minnesota Solvation Database [27–30], while the optimal aqueous solvation model [3] was applied without further adjustment. For both octanol compositions two models containing 1 or 2 free parameters were derived; the resulting four models were then used for predicting the SAMPL6 compound set log *P* values. After comparative analysis of these results we then discuss the relevance of individual tautomers in both phases, followed by an investigation into the origin of a remarkable outlier detected after experimental data have been revealed.

## Methods

### Theory

The (decadic) partition coefficient of a molecule is related to the Gibbs energy of transfer, $\Delta_{{{\text{trans}}}} G^{0}$, and therefore, via a thermodynamic cycle where the gas phase contributions cancel out, to the individual (standard) Gibbs energies, $G^{0}$, of the compound in the respective solvent (“wat” for water and “oct” for octanol) by

1 $$ \log P = - \frac{{\Delta_{trans} G^{0} }}{RT\ln 10} = \frac{{G_{wat}^{0} - G_{oct}^{0} }}{RT\ln 10} $$

where *R* is the molar gas constant and *T* is the temperature (298.15 K in this work). While the conceptual and theoretical basis for calculating these individual Gibbs energies is the same as in our previous works [3, 6, 14, 17], only neutral tautomers (“microstates”, subscript “*t*”) need to be considered whose Gibbs energies can be calculated via the discrete partition function approximation over conformations (“*c*”) by

2 $$ G_{t} = - RT\ln \sum\limits_{c} {\exp [ - G_{tc} /RT]} $$

Note that we here drop the superscript “0” indicating the standard state for simplicity, assuming infinite dilution conditions at an arbitrary formal concentration. The total Gibbs energy is then given by a similar partition function over the individual microstates as

3 $$ G = - RT\ln \sum\limits_{t} {\exp [ - G_{t} /RT]} $$

Within the EC-RISM formalism the Gibbs energy per conformation and per microstate is defined as

4 $$ G_{tc} = E_{tc}^{{{\text{sol}}}} + \mu_{tc}^{{\text{ex,corr}}} $$

where $E_{tc}^{{{\text{sol}}}}$ represents the electronic energy of a conformation in solution and $\mu_{tc}^{{\text{ex,corr}}}$ is the corrected excess chemical potential,

5 $$ \mu_{tc}^{{\text{ex,corr}}} = c_{\mu } \mu_{tc}^{{{\text{ex}}}} + c_{V} V_{tc}^{m} + c_{q} q, $$

ignoring entropic contributions from rotational and vibrational degrees of freedom. The uncorrected excess chemical potential, $\mu_{tc}^{{{\text{ex}}}}$, and the PMV $V_{tc}^{{\text{m}}}$ can be obtained from 3D RISM theory [31, 32], while the molecular net charge is a parameter that does not change between different tautomers or conformers of the same molecule. As only neutral forms were considered in this challenge, the parameter related to the net charge *q* (see Ref. [24] for a discussion on the possible physical origin of this term) does not play a role here. For octanol, we therefore trained only the parameters *c*_*µ*_ and *c*_*V*_ using experimental Gibbs energies of solvation that are computed by subtracting the gas phase energy of the molecule (*E*^vac^) from the EC-RISM Gibbs energy via

6 $$ \Delta_{{{\text{solv}}}} G^{0} = E^{{{\text{sol}}}} + \mu^{{\text{ex,corr}}} - E^{{{\text{vac}}}} $$

For water, we directly employed our optimal model derived earlier [3], which does not require scaling of the excess chemical potential term. We thus used only a single parameter (*c*_*V*_) for water and investigated the effect of using either one or two parameters for *n*-octanol, both as dry and wet phase.

### Computational details

The water model used in this work is identical to the most accurate SPC/E-based one used in the earlier SAMPL6 p*K*_a_ challenge [3], there denoted as “MP2/6–311+G(d,p)/*φ*_opt_”, i.e. from EC-RISM calculations using the exact electrostatic solute–solvent interactions obtained directly from the wave function.

For *n*-octanol the united atom model developed by DeBolt and Kollman [33] was used with an additional Lennard-Jones parameter of *σ* = 1.0 Å on the hydrogen atom of the hydroxyl group to avoid divergence of the RISM equations, similar to the modification used in the SPC/E water model. The octanol molecule was assumed to be fully extended and rigid (structure and parameters are provided as Online Resource 1). During the challenge a particle density of 3.82054 × 10^–3^ Å^−3^ and a dielectric permittivity of 9.86294 [34] were used for the dry octanol models while for the water-octanol mixture a dielectric permittivity of 9.1 and densities of 1.37473 × 10^–3^ Å^−3^ and 3.64253 × 10^–3^ Å^−3^ were chosen for the water and octanol sites, respectively, as estimated from the saturation molar fractions *x* by multiplying the molar mass-scaled *x* values with the wet octanol mass density (0.82883 g cm^−3^, *x*_wat_ of 0.274 [35]). During the post-challenge analysis we also prepared and tested alternative solvent properties using a more accurately extrapolated value for the dielectric permittivity of wet *n-*octanol of 8.41 [36] and the correct number densities of 1.3598 × 10^–3^ Å^−3^ and 3.65787 × 10^–3^ Å^−3^, corresponding to *n*-octanol with the experimental water mole fraction of 0.2705 [7]. The dielectric permittivity was estimated by fitting the experimental data for 303.15 K and 293.15 K with exponential functions and calculating the mean of the extrapolated values obtained at the experimental water mole fraction mentioned above. Data obtained under these conditions will be specifically marked in the Results section. The PMV was calculated via the 3D RISM total correlation function (*h*) route [31] using the 1D RISM estimate of the isothermal compressibility for water of 0.717062 × 10^9^ Pa^−1^, while for octanol the experimental compressibility of 0.761 × 10^9^ Pa^−1^ was used [37].

MNSOL structures for training of the *n*-octanol models were generated using the same workflow described in our SAMPL5 challenge paper [6], in this case using Gaussian 16 rev. B.01 [38] with tight convergence criteria and otherwise default settings during the QC optimization. For water 501 molecules were used for training while for *n*-octanol experimental values were available for 224 molecules. In the training phase up to five conformations were considered for each molecule by using a partition function approach where the free parameters occur in the exponents within the partition function expression, requiring non-linear regression by numerically minimizing the loss function

7 $$ \begin{gathered} L = \sum\limits_{{{\text{molecules}}}} {\left( { - RT\ln \sum\limits_{tc} {\exp [ - (E_{tc}^{{{\text{sol}}}} + c_{\mu } \mu_{tc}^{{{\text{ex}}}} + c_{V} V_{tc}^{{\text{m}}} )/RT]} } \right.} \\ \left. { - RT\ln \sum\limits_{{tc^{\prime}}} {\exp [ - E_{{tc^{\prime}}}^{{{\text{vac}}}} /RT]} - \Delta_{{{\text{solv}}}} G_{{\exp}}^{0} } \right)^{2} \\ \end{gathered} $$

where *c*′ in the second sum indicates that the vacuum conformations are not necessarily identical to those in *n-*octanol. For the SAMPL6 challenge molecules the initial force-field based structures (up to an energy threshold of 5 kcal mol^−1^) were further optimized at the B3LYP/6–311+G(d,p)/IEFPCM level of theory for both water and octanol, using the same settings described above, unlike the preceding SAMPL6 p*K*_a_ challenge stage [3] where at most the lowest two PCM optima were treated by EC-RISM.

3D RISM calculations utilized a periodic rectangular grid with 0.3 Å spacing and fixed cubic boxes of 128^3^ grid points. For water the PSE-2 closure was used, while due to convergence issues the PSE-1 (or Kovalenko-Hirata, KH) closure had to be used for the octanol calculations [12]. Convergence criteria, Lennard-Jones parametrization (GAFF 1.5, the nonbonded parameters are identical to version 1.4 [39]), and EC-RISM settings were chosen identical to our earlier work [3, 6], also applied here to octanol calculations.

## Results and discussion

### Gibbs energies of solvation in water and *n-*octanol

**Fig. 1:**
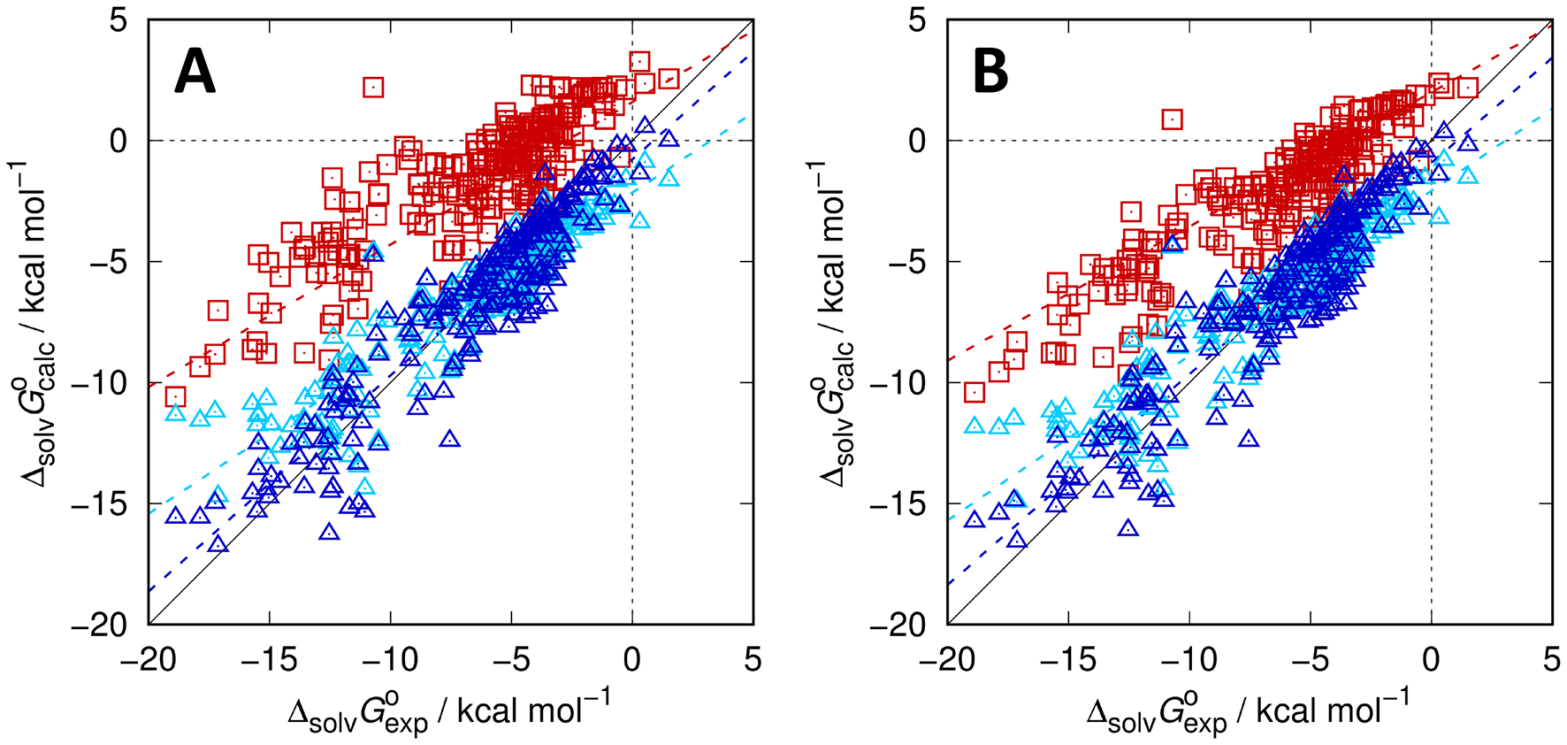
Calculated vs. experimental Gibbs energies of solvation in *n*-octanol for the MNSOL dataset [27] based on EC-RISM calculations for various *n*-octanol models: dry octanol (**A**) and wet octanol (**B**) using either a single (1-par, light blue triangles) or two parameters (2-par, dark blue triangles) in the trained correction. Uncorrected data is shown as red squares. Dashed lines indicate descriptive regression results. Optimized solution and gas phase structures are provided as Online Resource 2; calculated data, also split into separate components, are provided as Online Resource 3

The results of the training for the chosen water model, repeated here according to the optimal SAMPL6 p*K*_a_ setup [3], and the dry and wet *n*-octanol models under investigation are shown in Fig. 1 and Table 1, for the latter also including the optional scaling parameter for the excess chemical potential (“2-par”) besides the PMV-only correction (“1-par”). Statistical metrics and the adjustable parameters *c*_*µ*_, *c*_*V*_ and *c*_*q*_ (the latter only for water) are shown for each individual octanol model. It is observed that the results for the 2-parameter octanol models are generally comparable to those of the neutral compounds in water while the 1-parameter models perform slightly worse. The latter models exhibit a stronger deviation for molecules with lower Gibbs energies of solvation which is also visible in the significantly worse slope for those models. Somewhat counterintuitively, we also observe that the dry model performs slightly better in terms of the RMSE, while the MAE (mean absolute error) and MSE (mean signed error) indicate slightly better model balance in the wet case. If deduced only from the training set, all octanol models would be expected to perform reasonably well.

**Table 1:** Regression parameters of optimized EC-RISM-based Gibbs energy of solvation models (*c*_*q*_, *c*_*V*_ / kcal mol^−1^ Å^−3^, *c*_*q*_ / kcal mol^−1^ e^−1^) along with statistical metrics (root-mean-square error RMSE/kcal mol^−1^, mean absolute error MAE / kcal mol^−1^, mean signed error MSE / kcal mol^−1^, slope *m*′, intercept *b*′ / kcal mol^−1^, and coefficient of determination *R*^2^ from descriptive regression). For water, as taken from Ref. [3], separate metrics are reported for neutrals, anions, and cations in addition to the full MNSOL dataset

Solvent	RMSE	MAE	MSE	*m*′	*b*′	*R*^2^	*c*_*µ*_	*c*_*V*_	*c*_*q*_
Water									
All	2.04	1.43	− 0.26	1.00	− 0.35	1.00	–	− 0.10251	− 15.728
Neutrals	1.56	1.13	− 0.36	0.97	− 0.47	0.89	–	–	–
Anions	3.07	2.46	0.01	1.10	7.18	0.94	–	–	–
Cations	2.98	2.10	0.02	0.96	− 2.62	0.85	–	–	–
Octanol (dry)									
1-par	1.78	1.33	0.03	0.66	− 2.15	0.85	–	− 0.00799	–
2-par	1.48	1.14	− 0.08	0.89	− 0.78	0.87	1.33446	− 0.00609	–
Octanol (wet)									
1-par	1.73	1.31	− 0.01	0.68	− 2.08	0.85	–	− 0.01552	–
2-par	1.51	1.16	− 0.10	0.87	− 0.93	0.86	1.28924	− 0.01315	–

### SAMPL6 dataset: partition coefficients log *P*

**Fig. 2:**
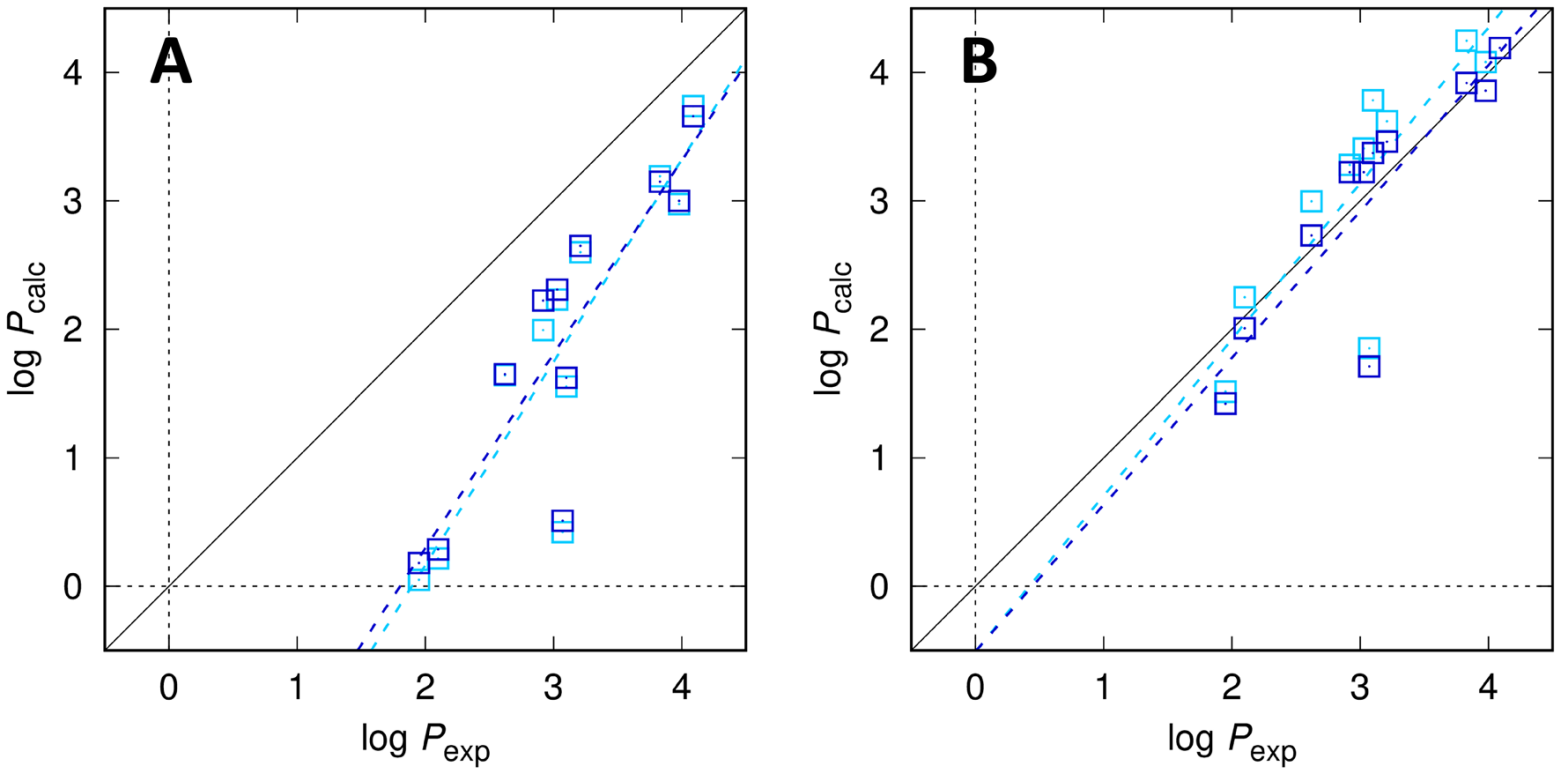
EC-RISM-derived vs. experimental log *P* values for the SAMPL6 log *P* dataset using either a single parameter (1-par) for the *n*-octanol model (**A**) or a two-parameter (2-par) *n*-octanol model (**B**). Data generated using dry/wet octanol are shown as light/dark blue squares, respectively. Optimized solution phase structures are provided as Online Resource 4; calculated data, also split into separate components, are provided as Online Resource 5

The resulting log *P* values from applying the various trained models to the molecules of the SAMPL6 challenge are shown in Fig. 2 and Tables 2 and 3. With an RMSE of 0.47 (rank 5 among all submissions, rank 2 among physics-based models) and MAE of 0.31 (rank 2 among all and physics-based submissions) for the best model (2-par, wet; submission ID *j8nwc*) our results are in line with the best performing models of this part of the SAMPL6 challenge (best RMSE and MAE: 0.38 and 0.31, respectively, for submission ID *hmz0n*). The ranking of the models also confirms our expectations with regards to the model quality: firstly, the models taking into account the water content of the organic phase perform slightly better than those ignoring it. Secondly, the octanol models using only a single parameter correcting for the partial molar volume perform significantly and systematically worse than the two-parameter octanol models. This confirms the training set’s trend, where the one-parameter models showed slopes deviating significantly from unity. Unlike the less clear expectation form the training phase, the dry models perform consistently worse than the wet models. This is of course reasonable from a physical point of view as the wet models contain relevant solute-water interactions and are capable of describing preferential solvation as a possible factor, but the overall performance is, surprisingly, still reasonable which indicates that the models are not dramatically overfitted. The quality loss of using only the PMV parameter (1-par models) is, however, more significant, which is somewhat unexpected, as a 2-parameter approach turned out to be unnecessary for water [6]. It is possible that the chosen united atom octanol model systematically underestimates the interactions between octanol and the solute, leading to the deviations seen in both the training set and the SAMPL6 challenge set of molecules.

**Table 2:** Individual experimental and corresponding predicted log *P* values for all models

	log *P*_exp_	Dry, 1-par	Wet, 1-par	Dry, 2-par	Wet, 2-par
SM02	4.09	3.74	3.66	4.56	4.19
SM04	3.98	2.97	3.00	4.08	3.86
SM07	3.21	2.60	2.65	3.62	3.46
SM08	3.10	1.55	1.62	3.78	3.37
SM09	3.03	2.23	2.31	3.41	3.22
SM11	2.10	0.22	0.29	2.25	2.01
SM12	3.83	3.19	3.15	4.25	3.92
SM13	2.92	1.99	2.22	3.28	3.22
SM14	1.95	0.05	0.18	1.51	1.42
SM15	3.07	0.42	0.51	1.85	1.71
SM16	2.62	1.64	1.65	3.00	2.73

Submission IDs for the individual submission are *2tzb0* (dry, 1-par), *rdsnw* (wet, 1-par), *qyzjx* (dry, 2-par), *j8nwc* (wet, 1-par)

**Table 3:** Statistical metrics for log *P* predictions (root-mean-square error RMSE, mean absolute error MAE, mean signed error MSE, slope *m*′, intercept *b*′, and coefficient of determination *R*^2^ from descriptive regression) for various models, encoded according to Table 2

Model	Submission ID	RMSE	MAE	MSE	*m*'	*b*′	*R*^2^
Dry, 1-par	*2tzb0*	1.38	1.21	− 1.21	1.58	− 2.99	0.79
Wet, 1-par	*rdsnw*	1.32	1.15	− 1.15	1.51	− 2.72	0.77
Dry, 2-par	*qyzjx*	0.54	0.45	0.15	1.22	− 0.51	0.73
Wet, 2-par	*j8nwc*	0.47	0.31	− 0.07	1.14	− 0.51	0.73

Furthermore, using the more accurate values for the density and dielectric constant does not significantly change the results obtained. The largest change is observed for the molecule SM12, for which the calculated log *P* increases from 3.92 to 3.93. Hence, the force field model impact is likely higher than small density uncertainties.

An interesting aspect to be derived from the present calculations concerns the so far unknown relevance of certain tautomers in both phases. While not explicitly part of this challenge, the tautomeric state of a compound in different environments, such as different solvents or a protein binding pocket in contrast to free aqueous solution is of general interest. In analogy to our calculation of microstate p*K*_a_ values during the first part of the SAMPL6 challenge [3] we therefore calculated the most stable tautomer in each phase and the relative tautomer stabilities of every other tautomer in that phase. Results are shown in Table 4. Throughout, the relative destabilization of the next higher tautomer compared to the most abundant one increases in octanol compared to water, the reasons for which require further investigation. Remarkably, we do not detect any tautomer shift or change of relative rankings upon changing the solvent environment. Again, this may be specific for this dataset and related to the large energetic gaps between dominant and next higher tautomer.

**Table 4:** Calculated Gibbs energies of the neutral microstates relative to the most favorable tautomer (microstate) of each compound for both solvents (in kcal mol^−1^)

Microstate	Water	Octanol (wet, 2-par)	Octanol (dry, 2-par)	Octanol (wet, 1-par)	Octanol (dry, 1-par)
SM02_micro002	0.00	0.00	0.00	0.00	0.00
SM02_micro003	5.16	5.57	5.66	5.65	5.71
SM02_micro007	6.18	8.86	8.80	10.30	10.40
SM04_micro003	0.00	0.00	0.00	0.00	0.00
SM04_micro004	8.45	9.81	9.74	10.68	10.76
SM04_micro009	11.10	11.72	11.78	12.15	12.24
SM07_micro002	8.97	10.59	10.61	11.63	11.78
SM07_micro003	6.75	7.97	8.00	8.34	8.41
SM07_micro004	0.00	0.00	0.00	0.00	0.00
SM08_micro008	10.26	24.63	24.61	32.59	33.52
SM08_micro010	5.69	6.05	6.56	4.70	4.89
SM08_micro011	0.00	0.00	0.00	0.00	0.00
SM09_micro002	6.79	9.55	9.45	11.45	11.57
SM09_micro003	0.00	0.00	0.00	0.00	0.00
SM09_micro011	5.60	6.02	6.09	6.46	6.55
SM11_micro005	0.00	0.00	0.00	0.00	0.00
SM11_micro028	7.14	8.07	8.21	8.46	8.61
SM11_micro029	14.81	17.69	17.68	18.81	18.93
SM11_micro030	26.91	34.04	34.12	36.10	36.40
SM12_micro002	4.73	5.21	5.32	5.35	5.43
SM12_micro011	5.76	8.48	8.42	10.04	10.14
SM12_micro012	0.00	0.00	0.00	0.00	0.00
SM13_micro005	0.00	0.00	0.00	0.00	0.00
SM13_micro007	6.23	6.28	6.31	6.69	6.76
SM13_micro009	8.01	10.72	10.51	12.78	12.84
SM14_micro001	0.00	0.00	0.00	0.00	0.00
SM14_micro005	28.76	37.41	37.02	41.99	42.23
SM15_micro001	9.24	19.80	18.80	26.68	26.76
SM15_micro002	0.00	0.00	0.00	0.00	0.00
SM16_micro002	0.00	0.00	0.00	0.00	0.00
SM16_micro003	12.41	13.39	13.61	12.68	12.79
SM16_micro007	6.75	11.48	11.49	13.61	13.93

Individual tautomer Gibbs energies in each solvent are provided as Online Resource 6. In contrast to the calculation of the partition coefficients where special treatment is not necessary, we here made sure that individual conformations undergoing a protonation shift during QC optimization were manually assigned to the correct microstate before evaluation of the partition function

### Post-submission analysis: correlation of errors with structural features

**Fig. 3:**
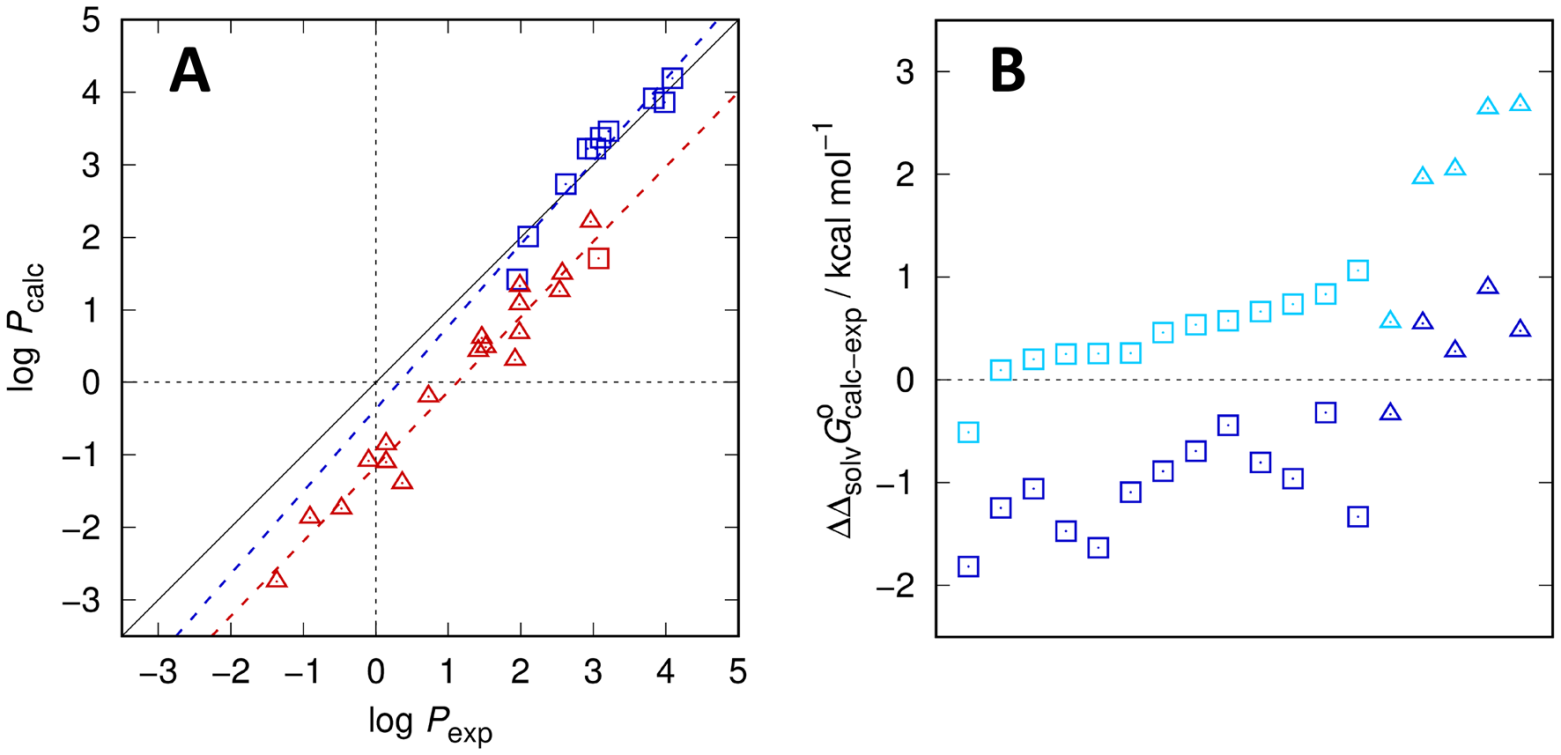
Calculated vs experimental log *P* of the combined SAMPL6 and MNSOL datasets (**A**) and errors in the solvation Gibbs energies of the MNSOL compounds in both solvents (**B**). In panel (**A**), SAMPL6 data are represented by squares, MNSOL data by triangles. Additionally, alcoholic compounds and their regression statistics are colored in red (*y* = 1.03 *x* − 1.16) while all other compound classes are shown in blue (*y* = 1.14 *x* − 0.37). In panel (**B**), aliphatic alcohols are depicted as squares while aromatic alcohols are depicted as triangles. Dark blue data points represent the errors of the solvation Gibbs energy in water, whereas light blue points refer to the errors of the solvation Gibbs energies in wet *n*-octanol, sorted in ascending *n*-octanol error order per group

A striking observation in the post-submission phase was the fact that only a single outlier, SM15, was responsible for the largest part of the error, omission of which would bring the RMSE down to 0.2. This compound is structurally very similar to the other molecules in this subset of the original SAMPL6 challenge, especially SM14, but it is unique in that it is the only species containing a hydroxyl group. Curiously, its log *P* is underestimated by many of the challenge participants (median error of ca. − 0.9 log *P* units, − 1.36 for our best model) in a way that is not seen for any other compound, as can be seen in the analysis files provided by the challenge authors [40]. This result led us to investigate the training dataset more closely during the post-submission phase, see Fig. 3. Comparing the calculated partition coefficients for all alcohols contained in the MNSOL Database for which solvation Gibbs energies in both water and *n*-octanol are available shows that a similar systematic offset is found for these molecules (Fig. 3a). The great benefit of the MNSOL data is that not only the partition coefficients but also the individual solvation Gibbs energies are available. Hence, we can dissect whether the error is due to insufficient accuracy in only one of the two phases. The errors in the solvation Gibbs energies revealed a mixed picture (Fig. 3b). For almost every aliphatic alcohol the prediction in *n*-octanol is better than that in water. Conversely, for almost every aromatic alcohol the water predictions are significantly better. The exception, *m*-cresol, is puzzling, especially since the experimental Gibbs energies of solvation of the three cresol isomers are within 0.6 kcal mol^−1^ for both solvents, while the predicted value fluctuates, only in octanol, by as much as 1.8 kcal mol^−1^. Still, the difference in the individual errors which gives rise to the constant deviation in the Gibbs energy of transfer and thus the log *P* remains almost constant across the entire range of compounds. This hints at a systematic problem with a model parameter that we have not touched in any of the preceding challenges, the dispersion-repulsion force field underlying the exact QC electrostatics. So far, we relied entirely on GAFF parameters [39] which might require further adjustment in order to make significant progress.

## Concluding remarks

In this challenge we were able to blindly predict the octanol–water partition coefficients of a set of small organic molecules to within 0.5 log *P* units. We achieved this by successfully reusing and improving older models and applying them to this new problem. In the earlier SAMPL6 p*K*_a_ challenge we already improved the water model by developing a new scheme for the treatment of exact electrostatics within EC-RISM in the post-submission phase. In the present challenge we thus focused on the *n*-octanol model. Modeling water-saturated octanol instead of dry octanol leads to a small, but consistent improvement of the predictive properties. Furthermore, unlike our findings for water, it appeared to be necessary to use a two-parameter model to achieve accurate solvation Gibbs energies after applying the PMV correction for *n*-octanol. Using more conformations per microstate does not lead to significantly improved results in all cases. For example, in the SAMPL6 p*K*_a_ challenge the inclusion of the second lowest conformation improved the total RMSE by only 0.02–0.08. However, it is not necessarily the case that the PCM minimum conformation is identical to the EC-RISM one, especially for large, flexible molecules with the potential for intramolecular interactions, so the inclusion of more than one conformer is still advisable.

There are multiple avenues for further improvement of the *n*-octanol model: like water, the octanol is modeled as a rigid body. While for water this ignores only the vibration of the molecule, which has been shown to be insignificant for such a small molecule [41], for a long, chain-like molecule such as *n*-octanol the significant torsional freedom of the carbon chain is lost. While unpublished results obtained in our group using intramolecular distribution functions extracted from molecular dynamics simulations do not indicate that this yields significantly improved solvation Gibbs energy predictions, these works were done before our participation in the SAMPL5 and SAMPL6 challenges which helped us improve the performance and reliability of EC-RISM. A reinvestigation, especially in combination with the wet octanol model is thus sensible.

A second area of improvement is the octanol model itself. While for water a variety of models have already been tested in our group, the octanol model by DeBolt and Kollmann [33] is the only one we have used in combination with EC-RISM. To find the best-performing model for this application, a comparison with other established models for the simulation of octanol such as the OPLS-UA or the TraPPE-UA models [42, 43] might be necessary.

Finally, the systematic deviation of molecules containing a hydroxyl group needs to be addressed in future works. The significantly smaller error in the transfer Gibbs energies and the correlation of the errors in the solvation Gibbs energies imply that the reasonable results obtained in this work relied on error cancellation between water and octanol terms to a certain extent. While it is necessary to establish this link on a larger and more diverse dataset, a reparametrization of certain atom types in the dispersion-repulsion (Lennard-Jones) force field might be the next step toward systematic EC-RISM performance improvement in general.

## Electronic supplementary material

Below is the link to the electronic supplementary material.

Supplementary file1 (ZIP 867 kb)
